# Necroptosis in primate luteolysis: a role for ceramide

**DOI:** 10.1038/s41420-019-0149-7

**Published:** 2019-02-11

**Authors:** Konstantin Bagnjuk, Jan Bernd Stöckl, Thomas Fröhlich, Georg Josef Arnold, Rüdiger Behr, Ulrike Berg, Dieter Berg, Lars Kunz, Cecily Bishop, Jing Xu, Artur Mayerhofer

**Affiliations:** 1 Biomedical Center Munich (BMC), Cell Biology, Anatomy III, Ludwig-Maximilians-University (LMU), Grosshaderner Strasse 9, Planegg, 82152 Germany; 2 Laboratory for Functional Genome Analysis LAFUGA, Gene Center, LMU, Feodor-Lynen Strasse 25, Munich, 81375 Germany; 3 Platform Degenerative Diseases, German Primate Center, Kellnerweg 4, Göttingen, 37077 Germany; 4 A.R.T. Bogenhausen, Prinzregentenstrasse 69, Munich, 81675 Germany; 5 Department Biology II, Division of Neurobiology, LMU, Grosshaderner Strasse 2, Planegg, 82152 Germany; 6 Division of Reproductive & Developmental Sciences, Oregon National Primate Research Center, Oregon Health & Science University, 505 NW 185th Avenue, Beaverton, Oregon 97006 USA

## Abstract

The corpus luteum (CL) is a transient endocrine organ, yet molecular mechanisms resulting in its demise are not well known. The presence of phosphorylated mixed lineage kinase domain-like pseudokinase pMLKL(T357/S358) in human and nonhuman primate CL samples (*Macaca mulatta* and *Callithrix jacchus*) implied that necroptosis of luteal cells may be involved. In *M. mulatta* CL, pMLKL positive staining became detectable only from the mid-late luteal phase onwards, pointing to necroptosis during regression of the CL. Cell death, including necroptosis, was previously observed in cultures of human luteal granulosa cells (GCs), an apt model for the study of the human CL. To explore mechanisms of necroptotic cell death in GCs during culture, we performed a proteomic analysis. The levels of 50 proteins were significantly altered after 5 days of culture. Interconnectivity analysis and immunocytochemistry implicated specifically the ceramide salvage pathway to be enhanced. *M. mulatta* CL transcriptome analysis indicated in vivo relevance. Perturbing endogenous ceramide generation by fumonisin B1 (FB1) and addition of soluble ceramide (C2-CER) yielded opposite actions on viability of GCs and therefore supported the significance of the ceramide pathway. Morphological changes indicated necrotic cell death in the C2-CER treated group. Studies with the pan caspase blocker zVAD-fmk or the necroptosis blocker necrosulfonamid (NSA) further supported that C2-CER induced necroptosis. Our data pinpoint necroptosis in a physiological process, namely CL regression. This raises the possibility that the primate CL could be rescued by pharmacological inhibition of necroptosis or by interaction with ceramide metabolism.

## Introduction

The corpus luteum (CL) forms after ovulation. Upon the ovulatoryluteinizing hormone (LH) surge granulosa and theca cells differentiate into large and small luteal cells, stop dividing and produce progesterone^1,2^. If conception occurs, chorionic gonadotropin (CG) stimulates survival of the CL and progesterone production. Otherwise the CL shuts down functionally and degenerates structurally.

Knowledge about the molecular events leading to functional and structural regression of the primate CL is limited. Low accessibility and significant differences in luteolytic events between primates and non-primate species may explain this lack of knowledge^3^. A fraction of the luteal cells undergo apoptosis in humans^4,5^, and involvement of autophagocytosis was suggested^6–8^. Both are immunologically silent events, yet other forms of cell death attract immune cells. Immune cells, for example, macrophages, appear to play an indispensable role in ovarian functions^9^ and CD11b positive macrophages invade the nonhuman primate CL during its regression and produce various cytokines and chemokines^10^.

Immune cell accumulation in the CL may be a consequence of necroptosis, a process recently suggested to occur in the regressing CL of cows^11^. Necroptosis is a combination of events, which include phosphorylation of receptor interacting protein kinase 1 (RIP1) and 3 (RIP3), formation of the necrosome, as well as phosphorylation of mixed lineage kinase domain-like pseudokinase (MLKL, at T357/S358) and its oligomerization to multimers including octamers^12,13^. Execution of necroptosis is associated with the typical morphological signs of necrosis^14^.

Fluidity of the cell membrane and lipid composition change during CL regression, and changes in sphingomyelin levels in combination with cholesterol levels are implicated in the loss of CL function^15^. It was shown that activation of the sphingomyelin pathway by Fas cell surface death receptor ligand (FASLG) and consequently production of ceramide led to cell death in bovine luteal cells^16^.

Sphingolipid metabolism is complex. Three distinct pathways of ceramide synthesis are known. First, the sphingomyelin degradation pathway leads to generation of ceramide by acid and neutral sphingomyelinases. This pathway is induced by FASLG, TNFα and oxidative stress^17,18^. Additionally, sphingolipids, especially ceramides, can be produced via *de novo* synthesis starting from serine and palmitoyl-CoA involving a cascaded reaction of 3-ketodihydrosphingosine reductase, dihydroceramide synthase and dihydroceramide desaturase in the endoplasmic reticulum^19^. Possible inducers of this pathway are heat stress, cannabinoids, chemotherapeutic agents and oxidized low density lipoprotein^20^. The third pathway is the ceramide salvage pathway. In late endosomes and lysosomes, sphingomyelin and complex sphingolipids are broken down to ceramide and sphingosine^21,22^. Sphingosine can then be reused to generate ceramide, which gives this pathway its name. Key enzymes of this pathway are acid sphingomyelinase (SMPD1), acid ceramidase (ASAH1) and acid β-glucosidase (GBA1). This pathway has a strong impact on intracellular signalling and has been linked to apoptosis in other cellular systems^23^. Recently, ceramide generation or its administration has also been linked to necroptosis^24,25^.

Human GCs are a unique model for the human CL. GCs stem from patients undergoing IVF and luteinize in culture. Investigations using this model led to the discovery of necroptosis in human GCs, in addition to apoptosis^26^. Inhibitors of MLKL (necrosulfonamid, NSA) and RIP1 (necrostatin-1, Nec-1) blocked necroptotic cell death. Evidence for in vivo relevance of this observation was obtained in ovarian sections of the rhesus macaque (*Macaca mulatta*) and the human, containing both follicles and the CL^26,27^. Strongest staining for pMLKL(T357/S358) was, however, found in CL samples.

Based on these observations we hypothesized that necroptosis is involved in primate luteolysis. To examine molecular mechanisms, we studied timed primate ovarian tissue, employed human IVF-derived GCs as a cellular model, and performed mass spectrometry and transcriptomic analysis. The results support that necroptosis occurs during luteolysis in primates, and pinpoint ceramide and the ceramide salvage pathway.

## Results

### Necroptosis occurs in the human and nonhuman primate CL

**Fig. 1:**
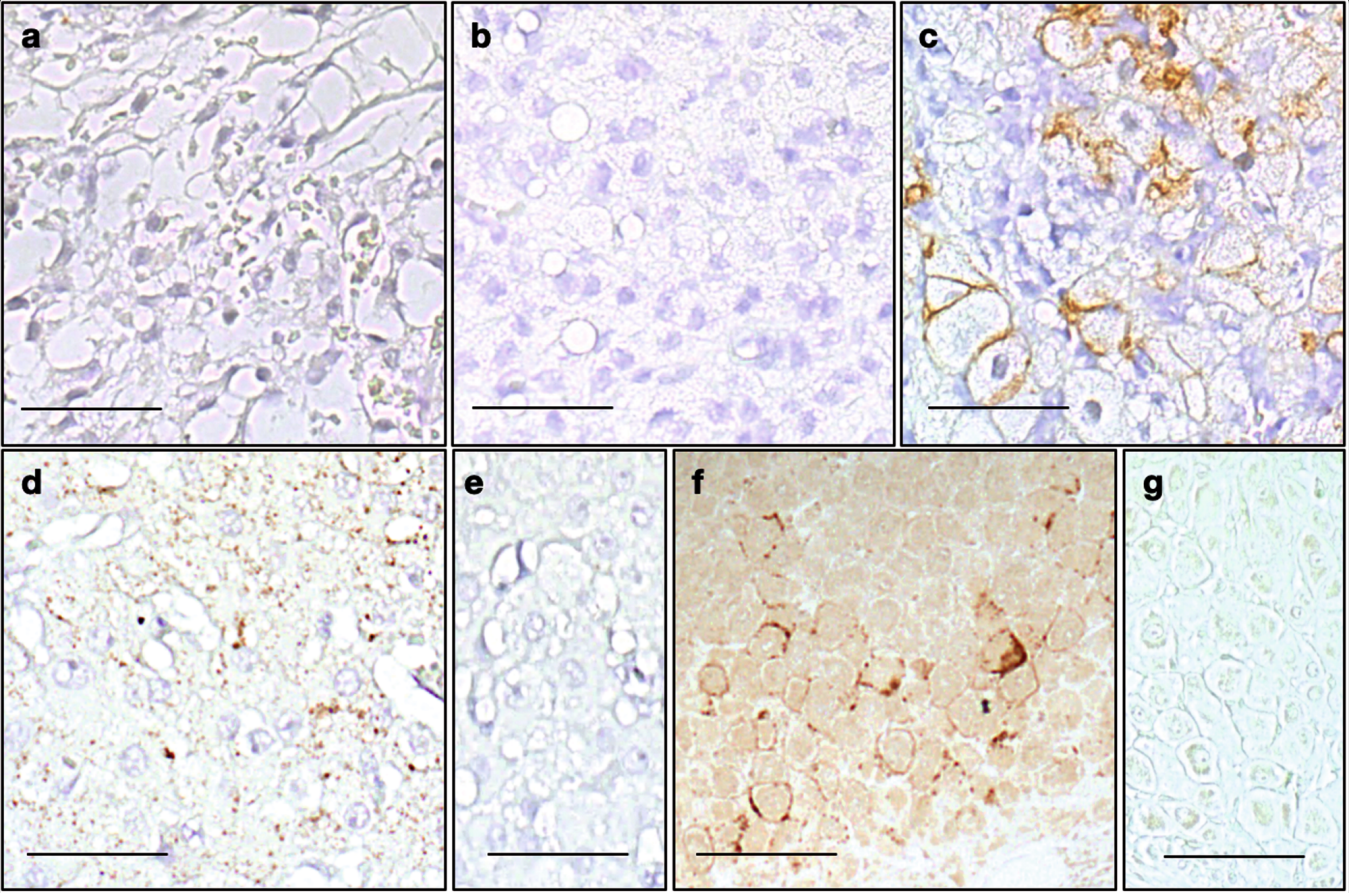
Immunohistochemical staining of pMLKL(T357/S358) in human and nonhuman primate corpus luteum. Immunohistochemical staining of pMLKL(T357/S358) in CL from different species. The upper panels depict macaque CL in early-luteal (3 day old, **a**) and mid-luteal phase (7 day old, **b**) as well as during regression (14 days old, **c**). Lower panels show pMLKL(T357/S358) staining of large luteal cells in sections of human (**d**) and marmoset (**f**) CL. Corresponding pre-absorption controls are shown (**e**, **g**). Scale bars: 50 µm. Representative images are shown

**Fig. 2:**
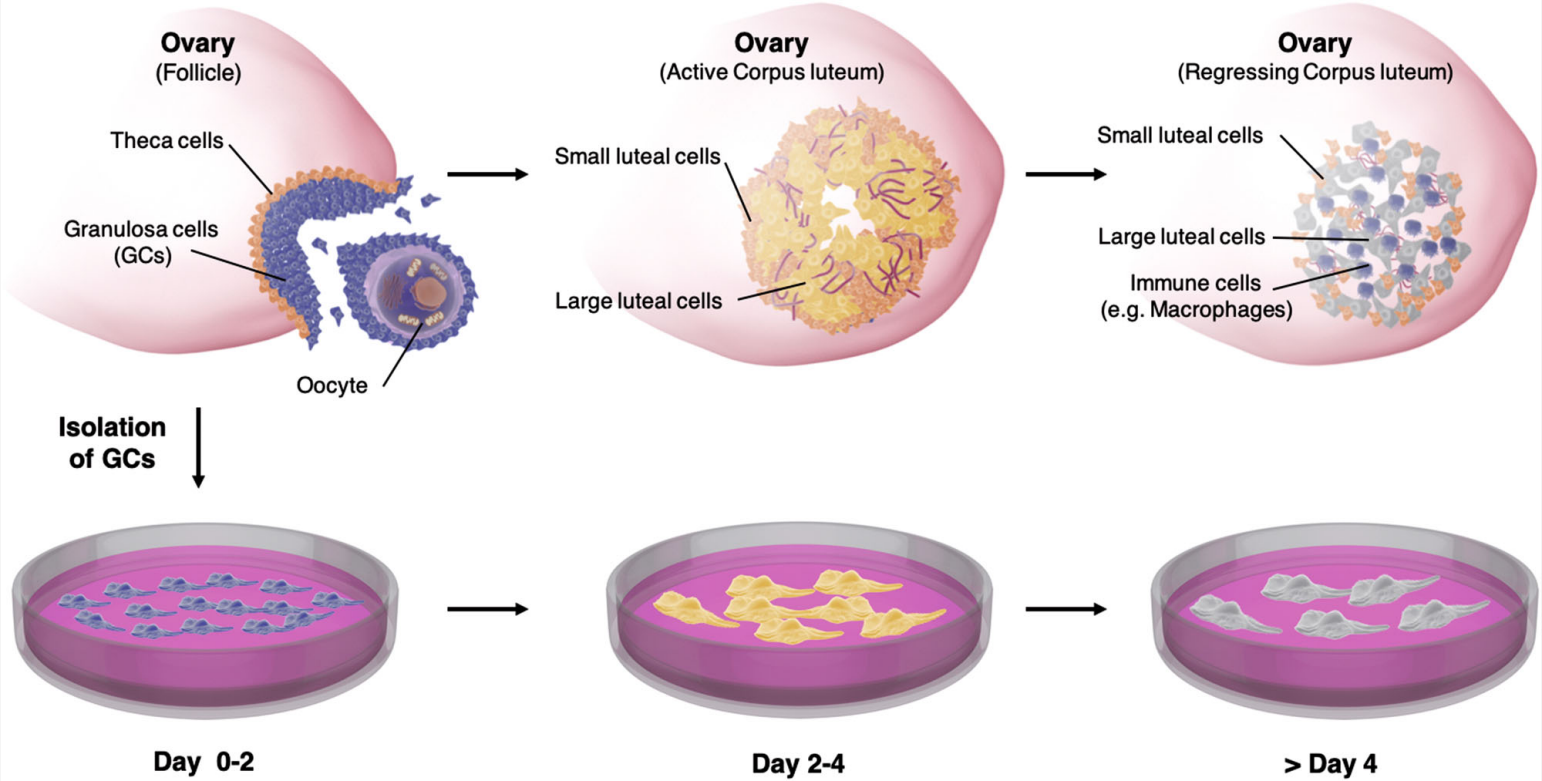
Schematic illustration of the events in the Corpus luteum (CL) and GC culture model. The upper panel depicts how the CL comes into existence upon ovulation. The residual theca cells differentiate into small luteal cells and mural GCs differentiate into steroidogenic, active large luteal cells. Together these cells form the CL, a highly vascularized, steroidogenic organ. During luteal regression, luteal cells stop producing hormones. The cells of the CL perish over time and the CL is invaded by immune cells, including macrophages. The whole luteal lifetime comprises approximately 14 days during a normal menstrual cycle. The lower panel depicts the fate of isolated GCs during IVF treatment. In culture, these cells differentiate to luteinized GCs and become steroidogenic within a couple of days. After approximately 4 days they become less active and eventually die

Immunohistochemical staining using anti-pMLKL(T357/S358) antibody provided evidence for necroptotic pathway activation in luteal cells of human and nonhuman primates (marmoset and macaque; Fig. 1). The immunoreactive cells were large luteal cells (Fig. 2). Preabsorption controls (Fig. 1e–g) were negative. CL samples from different stages were studied in macaques. While 3- and 7-day-old CL samples were negative for pMLKL(T357/S358) (Fig. 1a, b), cells positive for pMLKL(T357/S358) were evident in the 14 day-old CL (Fig. 1c).

### IVF-derived human GCs - a model for the human CL

IVF-derived human GCs luteinize and are considered a model for luteinized GCs of the CL (Fig. 2)^26^. We attempted to validate this model by using a proteome analysis of GCs cultured for 2 to 5 days. LC-MS/MS data from cultured GCs and literature data of human CL and in vivo-developed primate luteal cells stemming from the mid to late luteal phase were compared. The results indicated a high level of similarity. For example, the cholesterol side-chain cleavage enzyme (CYP11A1), a known luteal-cell marker, was highly expressed (24th most abundant of 3642 detected proteins) in cultured GCs^3^. Progesterone (P4) synthesis in the CL mainly requires three proteins next to CYP11A1, low density lipoprotein receptor (LDL-R), 3β-hydroxysteroid dehydrogenase (3β-HSD) and steroidogenic acute regulatory protein (StAR)^44^. All of these proteins were expressed in GC samples.

### Human granulosa cells undergo necroptosis during culture

**Fig. 3:**
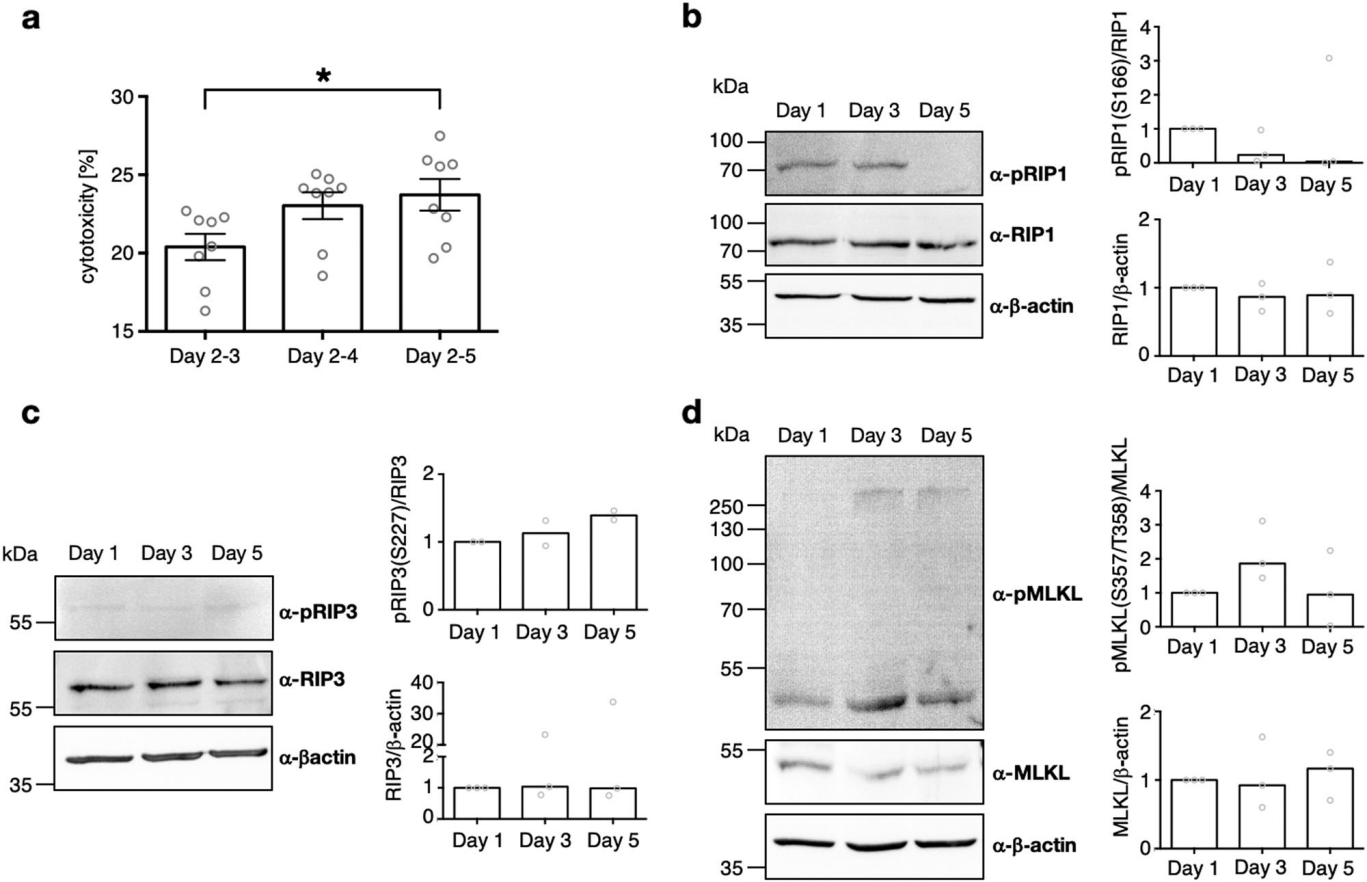
Signs of necro(pto)tic cell death during GC culture, evidenced by LDH assay and Western Blot. **a** Accumulative LDH levels starting of day 2 of GC culture. LDH levels were examined at day 3, 4 and 5 of GC culture using a colorimetric assay. Each data point represents one sample. One-way ANOVA with Dunett correction was carried out to examine statistical significance (*p < 0.05, *n* = 8, bars indicate SEM). **b**–**d** Representative Western Blots and relative quantification of results of GCs cultured for 1, 3, and 5 days. **b** pRIP1(S166), RIP1 and β−actin Western Blot. Quantification of band intensity was evaluated and expressed relative to RIP1 for the phosphorylated form and relative to β−actin for RIP1 (*n* = 3, median). **c** pRIP3(S227), RIP3 and β−actin Western Blot. Quantification of band intensity was evaluated and expressed relative to RIP3 for the phosphorylated form and relative to β−actin for RIP3 (*n* = 2, median). **d** pMLKL(T357/S358), MLKL and β−actin Western Blot. Quantification of band intensity was evaluated and expressed relative to MLKL for the phosphorylated form and relative to β−actin for MLKL (*n* = 3, median)

To examine necrotic events, we analysed LDH levels in medium. After medium change at day 2, we found a relative cytotoxicity of around 20 % (*n* = 8, Fig. 3a) in cells cultured until day 3 (i.e. 1 day of LDH accumulation), if compared to maximally possible LDH. LDH levels were rising over culture time (Fig. 3a). After 3 consecutive days of LDH accumulation, we found a significant difference compared to the samples analysed on day 3 (*p < 0.05).

We next studied proteins known to become phosphorylated during necroptosis (RIP1, RIP3, MLKL). Unphosphorylated forms of those were found at all timepoint during culture (Fig. 3b–d). In the beginning of necroptosis RIP1 is phosphorylated. Using anti-pRIP1(S166), we found the specific band, which was stronger on day 1 and 3 of culture if compared to day 5 (Fig. 3b). After RIP1 phosphorylation, RIP3 is recruited and phosphorylated at S227. An antibody against this phosphorylated peptide revealed a faint band at 62 kDa (Fig. 3c). Evaluation of band intensity showed a slight trend to more RIP3 being phosphorylated at late culture timepoints if compared to day 1. Finally, MLKL is recruited to form the necrosome and oligomerized pMLKL is the executor of necroptosis. Western blots with anti-pMLKL(T357/S358) showed stronger bands at day 3 of cultivation if compared to day 1 (Fig. 3d). Additionally, the oligomerized form was only detectable at day 3 onwards, indicating execution of necroptosis at these timepoints. The specificity of the anti-pMLKL(T357/S358) antibody was tested^27^. Taken together, the data indicate a trend of enhanced execution of necroptosis during culture time.

### Proteins associated with the ceramide salvage pathway were highly upregulated in GCs over culture period, as well as during in vivo-development of the CL

For GC samples from culture day 2 to 5, LC-MS/MS based proteomic analyses (*n* = 5 per day) were performed and a total of 3642 proteins were identified.

**Fig. 4:**
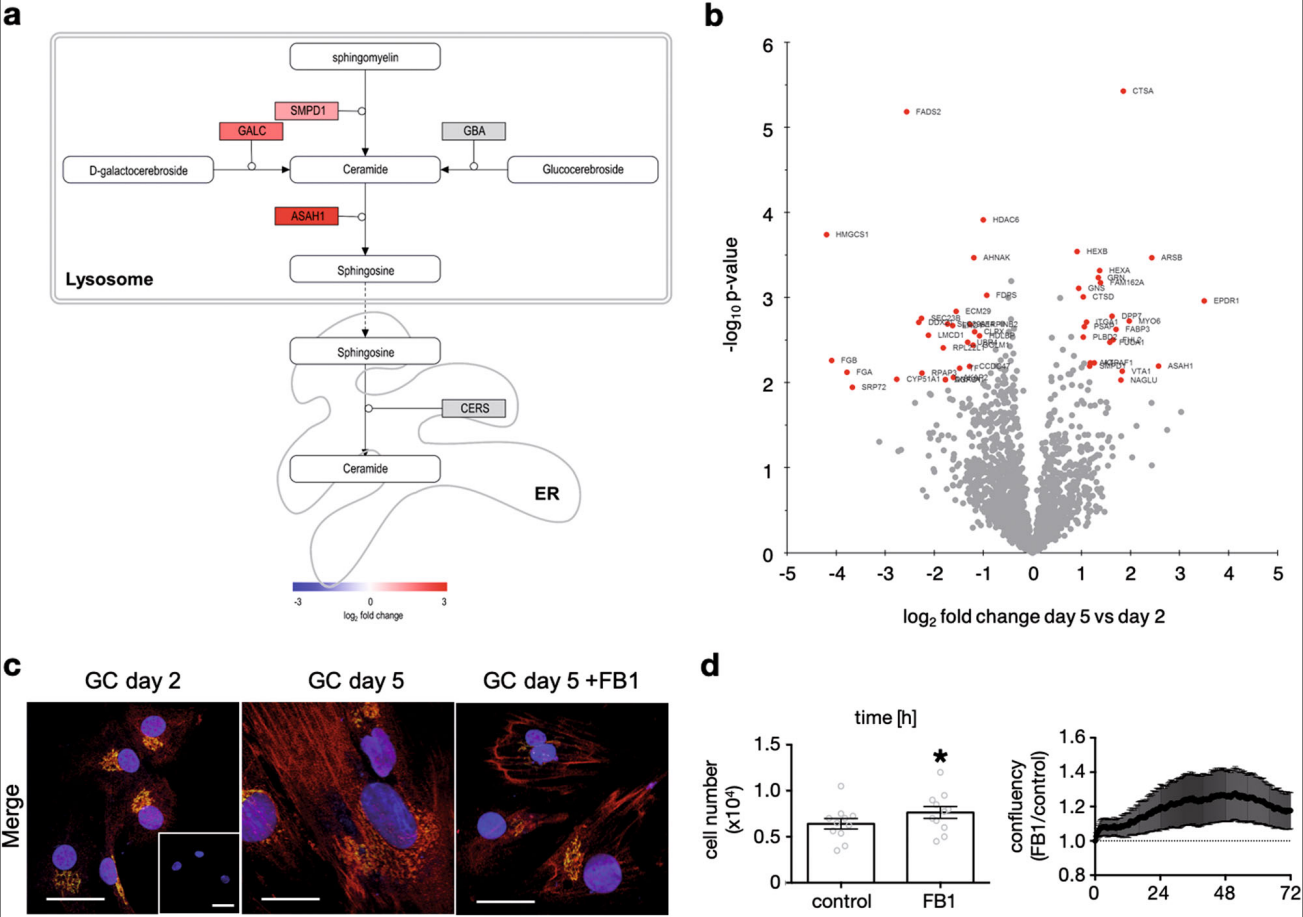
Proteomic analysis and verification of involvement of the endogenous ceramide system by FB1 blocking experiments. Levels of ceramide salvage pathway proteins increased during culture in human IVF-derived GCs. **a** Illustration of enzymatic reactions of ceramide within the salvage pathway in the lysosome, and ceramide synthesis in the endoplasmatic reticulum. Three different molecules get processed enzymatically to ceramide; Sphingomyelin by the acid sphingomyelinase, D-galactocerebroside by galactosylceramidase and glucocerebroside by acid beta-glucosidase. Ceramide then gets turned over by acid ceramidase to sphingosine, which can be processed back to ceramide in the endoplasmatic reticulum^61^. Proteins in grey were not significantly altered in abundance. Quantified proteins were colored with the shown gradient according to their log_2_ fold change between day 5 and day 2. **b** Volcano plot comparing day 5 with day 2 of human GC culture. Red marked proteins differed significantly in abundance after multiple testing correction. Table 2 shows the corresponding log_2_ fold changes as well as the *p* values. For each time point 5 GC samples were measured. **c** GCs, cultured on glass coverslips were fixed on day 2 or day 5 of culture. Cells were also stimulated with fumonisin B1 (FB1, 0.5 µM, 72 h) before fixation. Only merged pictures are shown, separated channels are available in Supplementary Fig. 4a. Immunocytochemical staining using anti-ceramide and anti-golgin97 antibodies revealed different intensities and cellular localization, depending on culture time and stimulation. Controls using rabbit IgG and mouse IgM are shown in inset of the first picture. Two experiments were performed and representative pictures are shown. Scale bars indicate 25 µm. **d** Cell numbers of GCs that were treated with FB1 (0.5 µM, *n* = 11) for 72 h are depicted in the bar diagram. On the right side, confluency data of FB1-stimulated stimulated GCs (0.5 µM, *n* = 6, a) are depicted, which were collected every 20 min and normalized to the corresponding solvent control. Paired Student’s *t* test was conducted to evaluate statistical significance (*p < 0.05); bars indicate SEM

Quantified proteins were prefiltered, based on *p* value and log_2_ fold change, and underwent a DAVID analysis to identify functional annotation clusters, which were enriched in day 5 compared to day 2. Three clusters were found (Table 1). The first cluster contained 7 proteins involved in cholesterol biosynthesis, which all showed lower abundancy at day 5. The second cluster included mainly translation initiation factors and translation associated proteins, which showed mostly small changes in abundancy. The third cluster contained 17 proteins, which were lysosome associated proteins. Most of these proteins are directly involved in the lysosomal ceramide salvage pathway and showed different degrees of raised abundance at day 5 ranging from log_2_ fold change 0.91 (HEXB) to 2.75 (GAA). An overview of the core pathway proteins and corresponding reactions is provided (Fig. 4a). For a detailed pathway see Supplementary Figure 1.

**Table 1:** DAVID clusters of proteome analysis

Protein name	Gene name	Uniprot accession	log_2_ fold change	*p* value
**Cluster 1: cholesterol biosynthesis**				
3-hydroxy-3-methylglutaryl-CoA synthase 1	HMGCS1	Q01581	−4.20	1.8E-04
7-dehydrocholesterol reductase	DHCR7	Q9UBM7	−0.81	2.6E-02
SWI/SNF related, matrix associated, actin dependent regulator of chromatin, subfamily d, member 3	SMARCD3	Q6STE5	−0.97	2.0E-02
Acetyl-CoA carboxylase alpha	ACACA	Q13085	−2.09	1.4E-02
Cytochrome P450 family 51 subfamily A member 1	CYP51A1	Q16850	−2.77	9.1E-03
Farnesyl diphosphate synthase	FDPS	P14324	−0.93	9.4E-04
Isopentenyl-diphosphate delta isomerase 1	IDI1	Q13907	−2.02	3.9E-02
**Cluster 2: translation initiation/ translation associated proteins**				
Eukaryotic translation initiation factor 3 subunit I	EIF3I	Q13347	−0.70	9.3E-03
Eukaryotic translation initiation factor 3 subunit J	EIF3J	O75822	−0.70	1.8E-02
Eukaryotic translation initiation factor 3 subunit K	EIF3K	Q9UBQ5	−1.63	1.6E-02
Eukaryotic translation initiation factor 3 subunit L	EIF3L	Q9Y262	−0.63	1.4E-02
Eukaryotic translation initiation factor 4A1	EIF4A1	P60842	−0.64	5.5E-03
Heat shock protein family B (small) member 1	HSPB1	P04792	−1.19	2.7E-02
Ribosomal protein L22-like 1	RPL22L1	Q6P5R6	−1.82	3.9E-03
Ribosomal protein L24	RPL24	P83731	−0.67	2.8E-02
Ribosomal protein S14	RPS14	P62263	−0.65	6.8E-03
**Cluster 3: lysosomal proteins**				
GM2 ganglioside activator	GM2A	P17900	1.39	1.7E-02
N-acetyl-alpha-glucosaminidase	NAGLU	P54802	1.80	9.4E-03
N-acylsphingosine amidohydrolase 1	ASAH1	Q13510	2.57	6.4E-03
Arylsulfatase B	ARSB	P15848	2.43	3.4E-04
Cathepsin A	CTSA	P10619	1.85	3.7E-06
Cathepsin D	CTSD	P07339	1.03	9.8E-04
Fucosidase, alpha-L- 1, tissue	FUCA1	P04066	1.58	3.4E-03
Galactosylceramidase	GALC	P54803	1.81	4.3E-02
Glucosamine (N-acetyl)-6-sulfatase	GNS	P15586	0.94	7.8E-04
Glucosidase alpha, acid	GAA	P10253	2.75	3.6E-02
Hexosaminidase subunit alpha	HEXA	P06865	1.37	4.8E-04
Hexosaminidase subunit beta	HEXB	P07686	0.91	2.9E-04
Neuraminidase 1	NEU1	Q99519	2.12	3.2E-02
Phospholipase B domain containing 2	PLBD2	Q8NHP8	1.04	2.9E-03
Prosaposin	PSAP	P07602	1.06	2.2E-03
Scavenger receptor class B member 2	SCARB2	Q14108	1.51	1.7E-02
Sphingomyelin phosphodiesterase 1	SMPD1	P17405	1.17	6.4E-03

Results of the DAVID annotation clustering. Three clusters were found to be enriched. Every cluster is shown with the corresponding proteins and the changes in abundancies

**Table 2:** 50 significantly differentially abundant proteins between day 2 and day 5 of GC culture

Protein name	Gene name	log_2_ fc	*p* value
Hydroxymethylglutaryl-CoA synthase, cytoplasmic	HMGCS1	−4.20	1.82E-04
Fibrinogen beta chain	FGB	−4.09	5.47E-03
Fibrinogen alpha chain	FGA	−3.78	7.55E-03
Signal recognition particle subunit SRP72	SRP72	−3.67	1.13E-02
Lanosterol 14-alpha demethylase	CYP51A1	−2.77	9.09E-03
Fatty acid desaturase 2	FADS2	−2.56	6.55E-06
Nucleolar RNA helicase 2	DDX21	−2.32	1.95E-03
Protein transport protein Sec23B	SEC23B	−2.26	1.75E-03
RNA polymerase II-associated protein 3	RPAP3	−2.26	7.72E-03
LIM and cysteine-rich domains protein 1	LMCD1	−2.12	2.76E-03
60 S ribosomal protein L22-like 1	RPL22L1	−1.82	3.90E-03
DnaJ homolog subfamily A member 1	DNAJA1	−1.78	9.18E-03
Arf-GAP domain and FG repeat-containing protein 1	AGFG1	−1.78	9.24E-03
Zinc transporter ZIP14	SLC39A14	−1.73	2.05E-03
EH domain-containing protein 1	EHD1	−1.63	2.14E-03
A-kinase anchor protein 2	AKAP2	−1.61	8.69E-03
Proteasome-associated protein ECM29 homolog	ECM29	−1.56	1.45E-03
Serotransferrin	TF	−1.48	6.79E-03
E3 ubiquitin-protein ligase UBR4	UBR4	−1.32	3.35E-03
Coiled-coil domain-containing protein 47	CCDC47	−1.28	6.43E-03
Plasminogen activator inhibitor 2	SERPINB2	−1.27	2.07E-03
Golgi membrane protein 1	GOLM1	−1.21	3.63E-03
Neuroblast differentiation-associated protein AHNAK	AHNAK	−1.19	3.39E-04
ATP-dependent Clp protease ATP-binding subunit clpX-like, mitochondrial	CLPX	−1.18	2.52E-03
Vigilin	HDLBP	−1.08	2.81E-03
Histone deacetylase 6	HDAC6	−1.00	1.22E-04
Farnesyl pyrophosphate synthase	FDPS	−0.93	9.37E-04
Beta-hexosaminidase subunit beta	HEXB	0.91	2.88E-04
N-acetylglucosamine-6-sulfatase	GNS	0.94	7.76E-04
Cathepsin D	CTSD	1.03	9.81E-04
Putative phospholipase B-like 2	PLBD2	1.04	2.91E-03
Prosaposin	PSAP	1.06	2.20E-03
Integrin alpha-1	ITGA1	1.10	1.94E-03
Sphingomyelin phosphodiesterase	SMPD1	1.17	6.37E-03
Adenylate kinase isoenzyme 1	AK1	1.17	5.85E-03
ATP synthase mitochondrial F1 complex assembly factor 1	ATPAF1	1.26	5.86E-03
Granulins	GRN	1.34	5.82E-04
Beta-hexosaminidase subunit alpha	HEXA	1.37	4.81E-04
Protein FAM162A	FAM162A	1.38	6.68E-04
Tissue alpha-L-fucosidase	FUCA1	1.58	3.36E-03
Dipeptidyl peptidase 2	DPP7	1.62	1.66E-03
Four and a half LIM domains protein 2	FHL2	1.64	3.14E-03
Fatty acid-binding protein, heart	FABP3	1.70	2.36E-03
Alpha-N-acetylglucosaminidase	NAGLU	1.80	9.35E-03
Vacuolar protein sorting-associated protein VTA1 homolog	VTA1	1.83	7.35E-03
Lysosomal protective protein	CTSA	1.85	3.72E-06
Unconventional myosin-VI	MYO6	1.97	1.89E-03
Arylsulfatase B	ARSB	2.43	3.38E-04
Acid ceramidase	ASAH1	2.57	6.39E-03
Mammalian ependymin-related protein 1	EPDR1	3.50	1.09E-03

Log_2_ fold change and *p* values of the proteins highlighted in the volcano plot shown in Fig. 2

Transcriptomic data of in vivo-developed macaque CL showed that 8 of 14 genes associated with the ceramide salvage pathway were significantly upregulated in the late stage CL relative to the early stage CL in macaques with log_2_ fold change ranging from 0.34 for arylsulfatase A (*ARSA*) to 1.51 for GM2 ganglioside activator (*GM2A*). ASAH1, GM2A, HEXA, HEXB, PSAP and SCARB2 were elevated in both the proteomics and the transcriptomics analysis (Supplementary Fig. 2, Supplementary Table 1). Validations employing real-time PCR revealed that the mRNA levels of *ASAH1*, *SMPD1*, *GBA* and *CERS*2 were likewise changed compared to the microarray data (data not shown).

The proteomics data were also filtered for q-values < 0.05 to identify highly significant differential abundance of proteins. The resulting 50 proteins were highlighted in a volcano plot (Fig. 4b). Several proteins were already found to be differentially abundant based on the DAVID enrichment analysis above, especially the lysosomal proteins. Granulins (GRN) were reported to have lysosomal activity and cytokine-like functions and were shown to be trafficked by prosaposin (PSAP) to the lysosome^45^. Both, GRN and PSAP were found to be highly abundant in cultured GCs on day 5. The high log_2_ fold change (-4.20) of the cytosolic hydroxymethylglutaryl-CoA synthase (HMGCS1) and the reduction of another protein associated with cholesterol synthesis, namely vigilin (also known as high density lipoprotein-binding protein, HDLBP, log_2_ fold change (-1.08)), became evident. The RNA binding protein vigilin is reported to protect cells from overaccumulation of cholesterol^46^. Collectively, these data point to the ceramide salvage pathway, and accumulation of its metabolic products ceramide and sphingosine, involved in cell death of in vitro cultured GCs and in vivo-developed CL.

### Cell studies using FB1 support a role of the ceramide pathway in GCs viability

The LC-MS/MS data in combination with the transcriptomic data in a nonhuman primate indicated an accumulation of ceramide or sphingosine in cultured GCs and in the regressing CL (Supplementary Fig. 2 and Supplementary Table 1). To further examine this possibility in vitro, immunocytochemical staining of ceramide species and the Golgi apparatus were carried out^35^. GCs at day 2 and day 5 of culture, as well as after 72 h of treatment with the known ceramide synthase blocker fumonisin B1 (FB1, 0.5 µM) were studied. At day 2, the ceramide staining was mainly localized to the Golgi apparatus, as shown by co-staining with anti-golgin97 (Fig. 4c). After 5 days of culture, cells had grown in size, which was indicated by the larger nuclei and Golgi apparatus. Ceramide staining intensity also increased. In addition to the Golgi apparatus, it was associated presumably with cell membranes of other cell compartments. As expected, FB1 treatment reduced ceramide staining intensity (Fig. 4c). These findings support the LC-MS/MS and transcriptomic data and show the significance of ceramide synthases in ceramide generation.

Next, we treated GCs with FB1 (0.5 µM) at day 1 of culture and determined cell confluency over a time period of 72 h (Fig. 4d). FB1-treatment increased confluency by 17.6 ± 11.1 % (*n* = 6, Fig. 4d, right diagram) compared to the solvent control. Furthermore, FB1 treatment significantly increased cell number (7.76 × 10^4^ ± 0.65 × 10^4^ cells, *n* = 11) compared to controls (6.41 × 10^4^ ± 0.57 × 10^4^ cells, *n* = 11, Fig. 4d, bar diagram). The data indicate that blocking ceramide synthases and therefore lowering ceramide levels has a positive effect on GC viability.

### A soluble ceramide analogue induced a form of necrotic cell death in human GCs

**Fig. 5:**
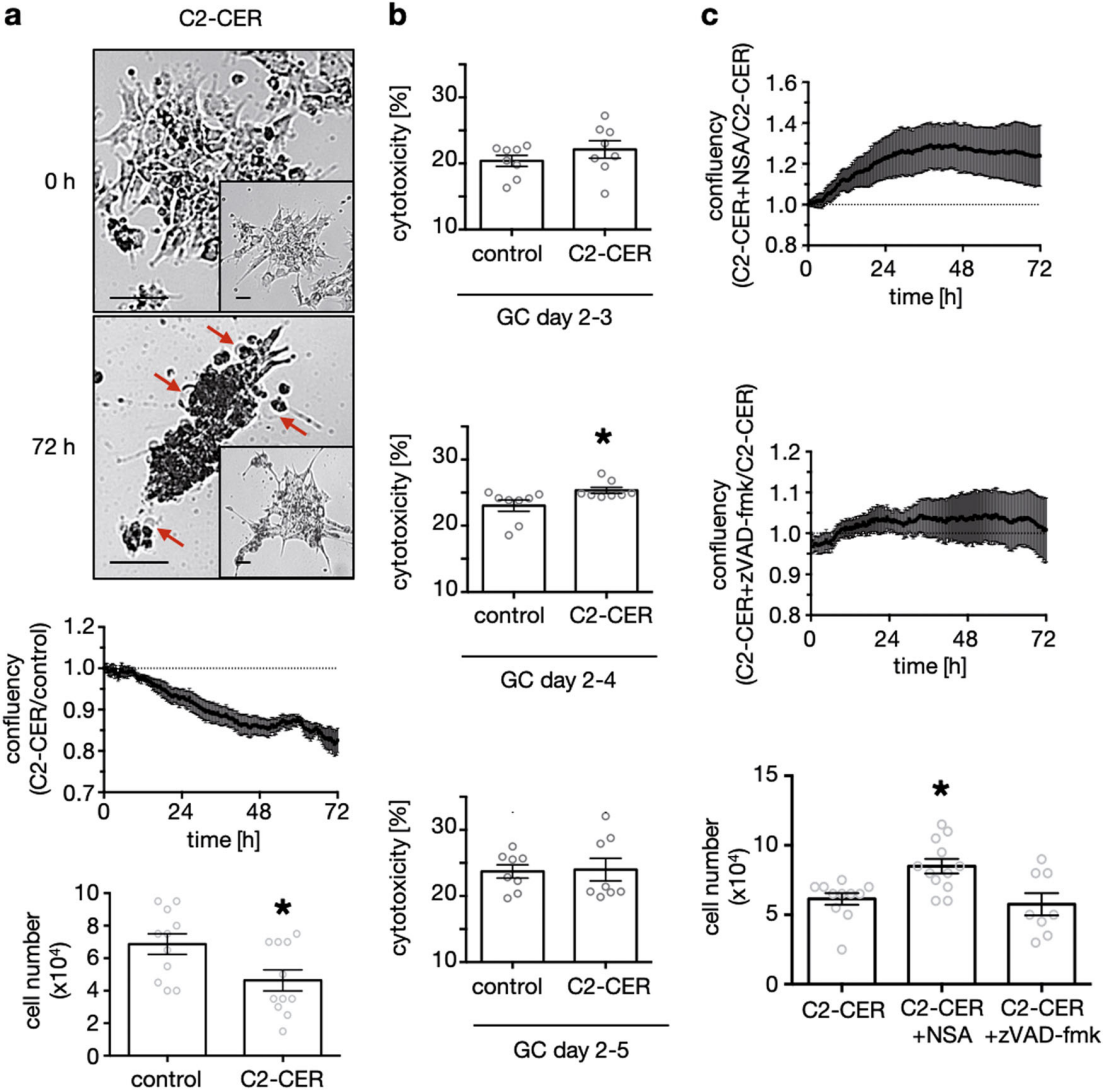
Exogenous administration of an ceramide derivative induced a form of necrotic cell death. **a** Representative pictures of C2-CER-stimulated GCs (50 µM) at the beginning (0 h) and in the end (72 h) of the stimulation are shown. Morphological signs of necrotic cell death e.g. ballooning are indicated by red arrows. Controls, which show less cell death are depicted within the inset, appropriate to the time points. Confluency data of C2-CER (50 µM, *n* = 9) stimulated GCs was collected every 20 min and normalized to the corresponding solvent control. Cell numbers of GCs, which were treated with C2-CER (50 µM, *n* = 11) for 72 h are depicted in the bar diagrams. **b** Colorimetric LDH Assay of GCs stimulated with C2-CER (50 µM) or solvent control between day 2 and 3 (upper diagram), day 2 and 4 (middle diagram) and day 2 and 5 (lower diagram). Cytotoxicity was evaluated according to the manufacturer’s recommendations. Each data point represents one sample (*n* = 8). Confluency data of GCs stimulated with C2-CER (50 µM, control) alone or in presence of NSA (20 µM, *n* = 6, a) or zVAD-fmk (20 µM, *n* = 5, b) were collected every 20 min for 72 h and normalized to the control group. **c** Cell numbers after 72 h of stimulation for the treatments C2-CER (50 µM, *n* = 11), C2-CER + NSA (50 µM, 20 µM, *n* = 12) and C2-CER + zVAD-fmk (50 µM, 20 µM, *n* = 8) are depicted. (**a**, **b**) paired Student’s *t*-test was conducted to evaluate statistical significance (*p < 0.05). (**c**) statistical significance was evaluated by testing against the control group using Kruskall–Wallis multiple comparison test (*p < 0.05). (**a**–**c**) bars indicate SEM

Cell studies with FB1 showed that endogenously produced ceramide has a negative effect on GC viability. To further examine the significance of ceramide in GC culture, we added the soluble ceramide analogue C2-ceramide (C2-CER, 50 µM) at day 1 or 2 of culture and evaluated confluency, cell number (Fig. 5a), as well as medium LDH levels (Fig. 5b). In the C2-CER-treated group we found significantly reduced confluency after 72 h by 17.5 ± 2.9 % (*n* = 9, Fig. 5a, x/y diagram) compared to the solvent control group. This was accompanied by an augmentation of typical morphological signs of necro(pto)tic cell death, e.g., ballooning (Fig. 5a, images). Additionally, 72 h stimulation with C2-CER significantly reduced cell number to 4.64 × 10^4^ ± 0.65 × 10^4^ (*n* = 11), compared to controls (6.86 × 10^4^ ± 0.64 × 10^4^, *n* = 11, Fig. 5a, bar diagram).

We measured accumulation of medium LDH after 24, 48 and 72 h, starting of day 2 of culture. LDH levels and subsequently relative cytotoxicity between day 2–3 were not significantly altered due to C2-CER (50 µM) stimulation (Fig. 5b, *n* = 8, upper diagram). After 48 h (Fig. 5b, *n* = 8, middle diagram), but not after 72 h (Fig. 5b, lower diagram), the effect of C2-CER on viability reached statistical significance. These results are in line with the confluency measurements (Fig. 5a), but the basal release of LDH is possibly superimposing the effect of C2-CER. Our studies on GCs using exogenously applied soluble C2-CER support the previous findings that ceramide induces a form of necrotic cell death in GCs.

### Necrosulfonamid but not the apoptosis inhibitor zVAD-fmk counteracted the effect of C2-CER

To analyse the types of cell death occurring in culture, we stimulated GCs with C2-CER (50 µM) alone or in combination with NSA (20 µM) or zVAD-fmk (20 µM) for 72 h, starting on day 1 or 2 of culture. The addition of NSA had a positive effect on confluency (121.3 ± 8.2 %, *n* = 6, Fig. 5c, upper diagram). Co-treatment with zVAD-fmk had no effect on confluency of C2-CER-treated GCs (102.4 ± 1.9 %, *n* = 5, Fig. 5c, middle diagram). Evaluation of cell numbers after a 72 h stimulation likewise showed a significant positive effect of NSA (8.50 × 10^4^ ± 0.53 × 10^4^ cells, *n* = 12) and no effect of zVAD-fmk (5.75 × 10^4^ ± 0.80 × 10^4^ cells, *n* = 8) in comparison to C2-CER-only treatment (6.14 × 10^4^ ± 0.42 × 10^4^ cells, *n* = 11, Fig. 5c, bar diagram).

As MLKL phosphorylation and oligomerization are the known terminal steps of necroptosis, we examined by western blot analyses whether the addition of C2-CER (50 µM, 72 h starting of day 1 or 2) is able to increase pMLKL(T357/S358). We detected bands corresponding to the monomeric and the oligomeric form of pMLKL in the C2-CER treated and the solvent group, yet without a significant difference (*n* = 5, Supplementary Fig. 3b). A high degree of variability became evident. Co-treatment of C2-CER (50 µM) and NSA (20 µM) yielded a significant reduction of pMLKL(T357/S358) levels, when compared to the group solely treated with C2-CER (*n* = 5, Supplementary Fig. 3c).

We also cultured GCs in the presence of NSA (20 µM, Supplementary Fig. 3a) for 72 h between day 2 and 5 and performed immunocytochemistry. As expected, we found increased ceramide staining over culture time, which unexpectedly was reduced upon addition of NSA (Supplementary Fig. 3a). To explore possible off-target effects, we conducted the same experiment using a blocker, which is known to have less off-target effects and acts as an upstream inhibitor of necroptosis by targeting RIP1, namely necrostatin 1 s (Nec-1s, 20 µM, Supplementary Fig. 4b)^47^. This experiment yielded comparable results supporting the hypothesis that ceramide generation and necroptosis are interlinked pathways.

## Discussion

Necrosis and necroptosis are generally linked to harmful or pathological processes^48^. Our cellular studies combined with the analysis of luteal samples indicate that necroptosis occurs in the CL of nonhuman primates and humans, as a physiological process linked to luteolysis, which is possibly fuelled by ceramide actions.

To date, very little is known about a physiological role of necro(pto)sis^49^. For example, during development of *C.elegans*, the linker cell helps to shape gonads in male worms and dies afterwards, lacking apoptosis marker but exhibiting morphological signs of necrosis^50^. In nonhuman primates, a functional study using ovarian follicle culture indicated involvement of necroptosis in follicular death^27^. A cell culture study in human GCs revealed spontaneously occurring necroptosis, which was further stimulated upon addition of a peptide (ARP) corresponding to a splice variant of acetylcholineesterase^26^. We now document phosphorylation of MLKL at T357/S358 in large luteal cells of regressing CL in macaques. Necroptotic cells were likewise identified in other primate species, including marmosets and humans, though the exact stages of the CL were not known. Furthermore, we assessed necroptotic cell death of GCs in a time course experiment, using pRIP1(S166), pRIP3(S277), pMLKL(T357/S358) and their unphosphorylated counterparts. We found necroptosis to occur over culture time. Due to pMLKL oligomerization at day 3 and 5 of culture we concluded that execution of necroptosis takes place on and after day 3 of culture. These results were supported by increasing LDH concentrations in culture medium over time.

As GCs differentiate in culture and also die over time, we reasoned that the culture of IVF-derived GCs is an apt model to explore possible mechanisms of necroptotic cell death related to the events in the CL. Results of a proteomic analysis of GCs allowed us to identify the ceramide salvage pathway. Many contributing enzymes were consistently upregulated, leading to the hypothesis that localization and/or quantity of sphingolipid species could change^20,51^. Comparison between the proteomic and the transcriptomic data indicated a high degree of similarity in protein and gene expression patterns between the two datasets, especially components involved in the ceramide salvage pathway, which indicated its physiological relevance. As both, GC necroptosis and elevated expression of ceramide salvage proteins take place during GC culture, we explored whether these pathways may be interlinked.

The salvage pathway may result in accumulation of two principle metabolites, ceramide and sphingosine^20^. Ceramide accumulation during necroptosis was shown before and was linked to *de novo* lipogenesis^52^. However, proteins associated with the synthesis of fatty acids were not changed in cultured GCs (ATP citrate lyase, ACLY; fatty acid synthase, FASN; fatty acid elongase1 and 5, ELOVL1 and 5) or were even strongly downregulated (acetyl-CoA carboxylase α, ACACA, log_2_FC = -1.74). Hence, we concluded that ceramide stems from the salvage pathway. An immunocytochemical approach endorsed the LC-MS/MS data, and ceramide levels increased with culture time. Addition of the ceramide synthase blocker FB1^31,32,53^ reduced  ceramide staining. FB1 also improved cell viability. Taken together, these results lead us to conclude that ceramide accumulation has a negative effect on GC viability by inducing cell death.

We also challenged GCs with a soluble, cell permeable ceramide analogue, C2-CER. It decreased cell viability. The change in confluency became evident during the first 48 h of imaging and was only marginally altered afterwards. Likewise, we found slightly but statistically significantly higher LDH levels due to C2-CER treatment albeit only after 48 h, a result which points at a necrotic form of cell death. Typical morphological signs of necro(pto)sis, including ballooning and cell burst, were evident in the C2-CER treated groups^54^.

The results obtained in GCs are in line with previous data^11,55,56^, which indicated that ceramide is associated with apoptosis or necroptosis in non-primate GCs. To further examine the forms of cell death in GCs, we tested the actions of NSA and zVAD-fmk. NSA is a known inhibitor of MLKL that covalently modifies Cys88, and subsequently blocks MLKL oligomerization and execution of cell death^57^.This blocker had an positive effect on confluency and cell number, which was lowered by C2-CER, whereas zVAD-fmk lacked such actions. While all these results point to the ability of C2-CER to induce necroptosis in GCs, Western Blot studies indicated that the known necroptosis executioner protein, i.e., phosphorylated MLKL, was not further elevated upon treatment. However, the addition of NSA reduced MLKL phosphorylation and lowered oligomerization of MLKL compared to C2-CER treated groups^12^. Human GCs are patient-derived primary cells and exhibit a large degree of variability^26^. It is possible that this fact contributes to the inability to decide from this type of experiment whether or to what degree the ongoing necroptotic events in GCs may be further enhanced by exogenous C2-CER.

Previous research showed that ceramide accumulation occurred downstream of RIP1 activation in TNFα-induced necroptosis^58^. However, there is also a link between ceramide production, general perturbation in cell metabolism and cell death^59^. We found that the addition of inhibitors targeting two steps in necroptosis, NSA and Nec-1s, both reduced ceramide staining. Clearly, while off-target actions of the drugs can not be ruled out, this may also indicate that ceramide production is complexly regulated in GCs and linked to necroptotic cell death.

In summary, results obtained in a cellular model and in vivo-developed CL from humans and nonhuman primates, indicate that necroptotic cell death contributes to the demise of the CL. This implicates necroptosis as a physiologically occurring event in the ovary. We suggest that the ceramide salvage pathway has a role in CL regression. Increased endogenous ceramide production is proposed as an inducer of this form of necrotic cell death in GCs. The cellular results also raise the possibility that the primate CL could be rescued by pharmacological inhibition of necroptosis or by modulation of ceramide metabolism. The applicability of such an approach in luteal-phase dysfunctions remains to be tested^60^.

## Materials and methods

### Culture and treatment of human IVF-derived GCs

The use of human IVF-derived GCs was approved by the ethics committee of the Ludwig-Maximilians University in Munich and each patient approved the use of cells. GCs were isolated from follicular fluid (FF), as previously described^28–30^. In brief, pooled FF from at least two patients was filtered (40 µm EASYstrainer, Grainer Bio-One, Kremsmünster, A), and the residuum was washed and backwashed with medium (1:1 DMEM/F12, Thermo Fisher Scientific, Waltham, MA, USA). Afterwards, cells were singularized through a 0.9 × 40 mm syringe (B.Braun Melsungen, Melsungen, GER) and centrifuged at 800 g for 3 min. Next, cell pellet was re-suspended in DMEM/F12 medium, supplemented with fetal calf serum (FCS, 10 %), penicillin (100 U/ml) and streptomycin (100 µg/ml), and counted using Neubauer chamber method. A total of 10^5^ cells were seeded per 35 mm^2^ plate (Sarstedt AG & Co. KG, Nümbrecht, GER) and cultivated at 37 °C and 5 % CO_2_. After 24 h of cultivation (day 1), non-adherent cells were removed by washing. For immunocytochemistry, cells were trypsinized at day 1 of culture and seeded onto glass coverslips in a 24 well plate (10^4^ cells/well, Sarstedt AG & Co. KG, Nümbrecht, GER). All experiments were carried out using cell culture medium.

For live cell experiments, GCs were stimulated with a synthetic, soluble and cell permeable ceramide analogue alone (C2-ceramide, 50 µM, Enzo Life Sciences Inc., Farmingdale NY, USA) or in combination with either the MLKL inhibitor necrosulfonamid (NSA, 20 µM, Cat. No. 5025) or the pan-caspase (apoptosis) inhibitor z-VAD-fmk (20 µM, Cat. No. 2163). Both inhibitors were ordered from Tocris Bioscience (Bristol, UK). Further, a widely used ceramide synthase inhibitor fumonisin B1 (FB1, 0.5 µM, Enzo Life Sciences Inc., Farmingdale NY, USA) was administrated to block endogenous ceramide generation^31,32^. All experiments were carried out for at least six times if not described otherwise.

### Confluency measurement and cell counting

Confluency of GCs between day 1 and day 5 of culture was measured for a period of 72 h, as described before^26^. For cell counting experiments, cells were rinsed with PBS post treatment, trypsinized and counted using the Neubauer chamber method. All experiments were carried out for at least eight times if not described otherwise.

### Immunohistochemistry

We immunohistochemically stained pMLKL(T357/S358) using the specific antibody (ab187091, Abcam, Cambridge, UK) to identify necroptosis in tissue sections of macaque, marmoset (*Callithrix jacchus*) and human ovaries. Samples from macaques were consecutive sections of those described previously^33^. Additional samples were provided by the Oregon National Primate Research Center, Oregon Health & Science University (Beaverton, OR, USA) collected during days 3–5 (early), days 7–8 (mid) and days 14–16 (late) after the midcycle LH surge in a previous study^34^. Marmoset samples are from the German Primate Center (Göttingen, GER) and were taken from the histological sample archive of the Platform Degenerative Diseases. The samples were fixed in Bouin’s solution and embedded in paraffin. All immunohistochemistry procedures were conducted as previously described^27^. In brief, sections were deparaffinized, antigens were retrieved using the HIER method, and endogenous peroxidase was blocked with H_2_O_2_ (3 in 10% methanol). Further, unspecific binding was prevented by incubation with 10 % goat serum in PBS. Positive antibody staining resulted from complexing of antigen bound primary antibody with biotinylated secondary antibody and avidin (ABC kit). Pre-absorption of anti-pMLKL(T357/S358) antibody using the respective peptide (ab206929, Abcam, Cambridge, UK) was done as previously described^27^. Immunohistochemical staining was carried out on three human CL samples, two marmoset CL samples and three timed series of macaque CL samples.

### Immunocytochemistry

To determine expression and localization of ceramide over culture time, we used a monoclonal anti-ceramide antibody (anti-CER, clone MID15B4, Enzo Life Sciences Inc., Farmingdale, NY, USA) in an immunocytochemical approach. This antibody detects different ceramide species including dihydroceramide, C16- and C24-ceramide^35^. Furthermore, anti-golgin97 antibody (Thermo Fisher Scientific, Waltham, MA, USA) was used to examine ceramide localization. Cells were stimulated at day 2 of culture with 0.5 µM FB1, 20 µM Necrostatin-1s (Nec-1s, a potent RIP1-kinase inhibitor) or 20 µM NSA for 72 h. After 5 days of culture and 72 h of stimulation, GCs were fixed in a 4% formaldehyde solution and permeabilized on ice using 0.2% Triton X-100 in PBS. To block unspecific binding, cells were incubated with 5 % goat serum in PBS. Specific immuno-decoration was achieved during 1.5 h incubation at room temperature. After three 5 min wash steps with 0.1 % Triton X-100 in PBS, a fluorophore-antibody conjugate was used to visualize specific antibody binding. For detection of anti-CER binding, Rhodamine conjugated F(ab’)_2_ fragment goat anti-mouse IgM (Jackson ImmunoResearch Inc., West Grove, PA, USA) was used (kindly provided by D. Dormann laboratory (Ludwig-Maximilians University, Department of Cell Biology, Munich, GER). Anti-golgin97 antibody was detected using Alexa488 conjugated donkey anti-mouse IgG (H + L) (Thermo Fisher Scientific, Waltham MA, USA). As controls, mouse serum IgG (Sigma-Aldrich, St. Louis, MI, USA) and IgM (Thermo Fisher Scientific, Waltham MA, USA) were used. Examination by confocal microscopy was conducted at the bioimaging core facility of the Biomedical Center (Ludwig Maximilians University, Munich, GER) using an inverted Leica SP8 microscope, equipped with lasers for 405, 488, 552 and 638 nm excitation. Images were acquired with a HC PL APO 63 × /1.40 oil objective. Fluorescence was recorded with hybrid photo detectors (HyDs), and DAPI with a conventional photomultiplier tube. All experiments were carried out for at least three times.

### LDH assay

Pierce LDH Cytotoxicity Assay Kit (PI, Thermo Fisher Scientific, Waltham, USA) was carried out as recommended by the manufacturer and described previously^26^. In brief, GCs were isolated and pools of at least 2 patients were cultured for 24 h. At day 1 of culture the cells were trypsinized and seeded at 10^4^ cells/well in 96-well plates. At day 2 of culture growth medium was changed and cells were treated with C2-CER (50 µM) or solvent control. After 1, 2 or 3 days of stimulation LDH levels were measured using a microplate reader (FLUOStar Optima, BMG Labtech, Ortenberg, GER). To calculate cytotoxicity a serum control, serum free control and controls treated with lysis buffer before measurement (max LDH) were included.

### Protein isolation and western blot

Protein isolation and Western Blot were conducted as previously described^26,27^. In brief, GCs were lysed after 1 to 5 days of culture using RIPA buffer containing protease and phosphatase inhibitors (PI, Thermo Fisher Scientific, Waltham, USA). A total of 10 µg protein per lane was loaded on a 12 % SDS gel and run under constant current (30 mA/gel). After blotting (100 V, 65 min) and blocking with 5 % non-fat dry milk in Tris-buffered saline with Tween 20 (TBS-T, 50 mM Tris-HCl, 150 mM NaCl, 0.1 % Tween 20, pH 7.4), anti-pRIP1(S166), anti-pRIP3(S227) both from Cell Signaling Technology (Danvers, MA, USA) and anti-pMLKL(T357/S358) antibodies were administered to decorate these phosphorylated proteins. To visualize specific binding, HRP-conjugated goat anti-rabbit antibody (Jackson ImmunoResearch Inc., West Grove, PA, USA) was used. As loading controls, anti-RIP1, anti-RIP3 (both from Cell Signaling Technology, Danvers, MA, USA), anti-MLKL (ab184718, Abcam, Cambridge, UK) and anti-β-actin (A5441, Sigma-Aldrich, St. Louis, MI, USA) antibodies were used. Preabsorption of anti-pMLKL was previously published^27^. All experiments were carried out five times if not described otherwise.

### Protein mass spectrometry (LC-MS/MS)

GCs cultured for 2 to 5 days were analyzed using a label-free approach. LC-MS/MS was performed as previously described^36^. In brief, 2.5 µg total proteins were reduced using DTT, alkylated and digested at 37 °C with LysC for 4 h followed by trypsin overnight. LC-MS/MS was performed with an Ultimate 3000 RSLC chromatography system coupled to a 5600^+^ mass spectrometer.

Raw files were processed using MaxQuant (version: 1.5.8.3) and default settings with the following exceptions: (a) label-free quantification was set to on; (b) “LFQ minimum ratio count” was set to 1; and (c) the “match between runs” feature was turned on. As databases, the human Swiss-Prot subset (Release 06/2017) and the common contaminants from MaxQuant were used^37^. For protein Identification, the data were searched with a target decoy approach resulting in a < 1% False Discovery Rate (FDR). The mass spectrometry data were submitted to the ProteomeXchange Consortium via the PRIDE partner repository (identifier PXD010658). Statistical analysis was done with Perseus (version 1.6.0.7). The samples were first grouped based on their day of cultivation. For pair-wise comparisons, a minimum of 4 values in at least one group were required. The filtered and imputed data were used to calculate log_2_ fold changes with a two-sided Welch’s *t*-test. Multiple Testing correction was performed using the Permutation-based FDR (5% FDR) which is based on calculation of q-values and provided with Perseus. Proteins with a q-value < 0.05 and a log_2_ fold change > |0.6| were considered significantly different in abundance. For the “DAVID Functional Annotation Clustering”, proteins were filtered less stringent with a *p* value < 0.05 and a log_2_ fold change > |0.6|^38,39^. This list was then analyzed with the DAVID online tool to build annotation based clusters according to the GO terms (molecular function, biological process and cellular component) and the reactome pathway database. Only clusters where at least one annotation component had a corrected *p* value (Benjamini) < 0.01 and an enrichment score > 2.5 were seen as significantly enriched.

Pathway illustrations were done with pathvisio^40,41^. The volcano plot was performed with Microsoft Excel (Redmond, USA) using the results from the Welch’s *t*-test mentioned above.

### Microarray and real-time PCR

This was a retrospective study assessing mRNA levels of target genes in rhesus macaque CL samples collected during day 3–5 (early) and day 14–16 (late) after the midcycle LH surge in a previous study^42^. The normalized and log transformed (base 2) microarray data were downloaded from the NCBI Gene Expression Omnibus repository (https://www.ncbi.nlm.nih.gov/gds) Dataset Series #GSE10367^42^.The target genes searched were *ARSA*, *ASAH1*, *CTSA*, *GALC*, *GBA*, *GLA*, *GLB1*, *GM2A*, *HEXA*, *HEXB*, *NEU1*, *PSAP*, *SCARB2*, and *SMPD1*. The RNA samples used for microarray analysis were used to synthesize cDNA, as described previously^42^. Real-time PCR was performed using TaqMan Gene Expression Assays (Thermo Fisher Scientific, Waltham, MA, USA) and Applied Biosystems 7900HT Fast Real-time PCR System (Thermo Fisher Scientific, Waltham, MA, USA), as previously described^43^, for selected genes including *ASAH1* (Assay ID: Hs00602774_m1), *CERS2* (Assay ID: Hs00371958_g1), *GBA* (Assay ID: Hs00986836_g1), and *SMPD1* (Assay ID: Hs003679347_g1). Mitochondrial ribosomal protein S10 (*MRPS10*) served as the internal control.

### Statistical analysis

Graphs were constructed in Prism 6 running under Mac OS X, whereby statistical analysis of cell counts, LDH Assays, Western Blot and gene expression were done using the same program and two-sided Student’s *t* test. LDH Assay with more than two groups were analysed using One-way ANOVA with Dunett correction. Cell count experiments with more than two independent groups were analysed using Kruskall–Wallis multiple comparison test. *P* values < 0.05 were considered as significant. The microarray transcriptome analysis was performed using the GeneShifter (VizX Labs, Seattle, WA, USA) software and the Affymetrix Expression Console, as described previously^42^.

## Supplementary information

supplementary files

## References

[1] Richards JS, Russell DL, Ochsner S, Espey LL (2002). Ovulation: new dimensions and new regulators of the inflammatory-like response. Annu. Rev. Physiol..

[2] Green C, Chatterjee R, McGarrigle HH, Ahmed F, Thomas NS (2000). p107 is active in the nucleolus in non-dividing human granulosa lutein cells. J. Mol. Endocrinol..

[3] Devoto L (2009). The human corpus luteum: life cycle and function in natural cycles. Fertil. Steril..

[4] Shikone T (1996). Apoptosis of human corpora lutea during cyclic luteal regression and early pregnancy. J. Clin. Endocrinol. Metab..

[5] Morales C (2000). Different patterns of structural luteolysis in the human corpus luteum of menstruation. Hum. Reprod..

[6] Mizushima N (1998). A protein conjugation system essential for autophagy. Nature.

[7] Grzesiak M, Knapczyk-Stwora K, Słomczyńska M (2016). Induction of autophagy in the porcine corpus luteum of pregnancy following anti-androgen treatment. Can. J. Physiol. Pharmacol..

[8] Bulling A (2000). Identification of an ovarian voltage-activated Na + -channel type: hints to involvement in luteolysis. Mol. Endocrinol..

[9] Wu R, Van der Hoek KH, Ryan NK, Norman RJ, Robker RL (2004). Macrophage contributions to ovarian function. Hum. Reprod. Update.

[10] Bishop CV (2017). Changes in immune cell distribution and their cytokine/chemokine production during regression of the rhesus macaque corpus luteum. Biol. Reprod..

[11] Hojo T (2016). Programmed necrosis - a new mechanism of steroidogenic luteal cell death and elimination during luteolysis in cows. Sci. Rep..

[12] Huang, D. *et**al*. The MLKL channel in necroptosis is an octamer formed by tetramers in a dyadic process. *Mol. Cell Biol.* **37**, 10.1128/mcb.00497-16 (2017). 10.1128/MCB.00497-16 PMC5311246 27920255

[13] Galluzzi L (2018). Molecular mechanisms of cell death: recommendations of the Nomenclature Committee on Cell Death 2018. Cell Death & Differ..

[14] Linkermann A, Green DR (2014). Necroptosis. N. Eng. J. Med..

[15] Carlson JC, Buhr MM, Riley JC (1989). Plasma membrane changes during corpus luteum regression. Can. J. Physiol. Pharmacol..

[16] Pru JK, Hendry IR, Davis JS, Rueda BR (2002). Soluble Fas ligand activates the sphingomyelin pathway and induces apoptosis in luteal steroidogenic cells independently of stress-activated p38MAPK. Endocrinology.

[17] Kim MY, Linardic C, Obeid L, Hannun Y (1991). Identification of sphingomyelin turnover as an effector mechanism for the action of tumor necrosis factor alpha and gamma-interferon. Specific role in cell differentiation. J. Biol. Chem..

[18] Goldkorn T (1998). H2O2 acts on cellular membranes to generate ceramide signaling and initiate apoptosis in tracheobronchial epithelial cells. J. Cell. Sci..

[19] Menaldino DS (2003). Sphingoid bases and de novo ceramide synthesis: enzymes involved, pharmacology and mechanisms of action. Pharmacol. Res..

[20] Kitatani K, Idkowiak-Baldys J, Hannun YA (2008). The sphingolipid salvage pathway in ceramide metabolism and signaling. Cell. Signal..

[21] Smith ER, Merrill AH (1995). Differential roles of de novo sphingolipid biosynthesis and turnover in the “burst” of free sphingosine and sphinganine, and their 1-phosphates and N-acyl-derivatives, that occurs upon changing the medium of cells in culture. J. Biol. Chem..

[22] Ogretmen B (2002). Biochemical mechanisms of the generation of endogenous long chain ceramide in response to exogenous short chain ceramide in the A549 human lung adenocarcinoma cell line. Role for endogenous ceramide in mediating the action of exogenous ceramide. J. Biol. Chem..

[23] Takeda S, Mitsutake S, Tsuji K, Igarashi Y (2006). Apoptosis occurs via the ceramide recycling pathway in human HaCaT keratinocytes. J. Biochem..

[24] Mizumura K (2018). Sphingolipid regulation of lung epithelial cell mitophagy and necroptosis during cigarette smoke exposure. FASEB J.: Off. Publ. Fed. Am. Soc. Exp. Biol..

[25] Zhang X (2018). Ceramide nanoliposomes as a MLKL-dependent, necroptosis-inducing, chemotherapeutic reagent in ovarian cancer. Mol. Cancer Ther..

[26] Blohberger, J. *et**al*. Readthrough acetylcholinesterase (AChE-R) and regulated necrosis: pharmacological targets for the regulation of ovarian functions? *Cell Death Dis.* **6**, e1685 (2015). 10.1038/cddis.2015.51 PMC4385929 25766324

[27] Du Y, Bagnjuk K, Lawson MS, Xu J, Mayerhofer A (2018). Acetylcholine and necroptosis are players in follicular development in primates. Sci. Rep..

[28] Mayerhofer A (1999). Functional dopamine-1 receptors and DARPP-32 are expressed in human ovary and granulosa luteal cells in vitro1. J. Clin. Endocrinol. Metab..

[29] Mayerhofer A, Föhr KJ, Sterzik K, Gratzl M (1992). Carbachol increases intracellular free calcium concentrations in human granulosa-lutein cells. J. Endocrinol..

[30] Mayerhofer A, Sterzik K, Link H, Wiemann M, Gratzl M (1993). Effect of oxytocin on free intracellular Ca2 + levels and progesterone release by human granulosa-lutein cells. J. Clin. Endocrinol. & Metab..

[31] Wang E, Norred WP, Bacon CW, Riley RT, Merrill AH (1991). Inhibition of sphingolipid biosynthesis by fumonisins. Implications for diseases associated with Fusarium moniliforme. J. Biol. Chem..

[32] Kim, S. H., Singh, M. P., Sharma, C. & Kang, S. C. Fumonisin B1 actuates oxidative stress-associated colonic damage via apoptosis and autophagy activation in murine model. *J. Biochem. Mol. Toxicol*. e22161 (2018). 10.1002/jbt.22161. Epub ahead of print. 10.1002/jbt.22161 29785744

[33] Kunz L (2002). Ca2 + -activated, large conductance K + channel in the ovary: identification, characterization, and functional involvement in steroidogenesis. J. Clin. Endocrinol. Metab..

[34] Xu F, Stouffer RL (2009). Existence of the lymphatic system in the primate corpus luteum. Lymphat. Res. Biol..

[35] Cowart LA, Szulc Z, Bielawska A, Hannun YA (2002). Structural determinants of sphingolipid recognition by commercially available anti-ceramide antibodies. J. Lipid Res..

[36] Schmid, N. et al. Characterization of a nonhuman primate model for the study of testicular peritubular cells - comparison with human testicular cells. *Mol. Hum. Reprod.*10.1093/molehr/gay025 (2018). 10.1093/molehr/gay025 29846669

[37] Cox J, Mann M (2008). MaxQuant enables high peptide identification rates, individualized p.p.b.-range mass accuracies and proteome-wide protein quantification. Nat. Biotechnol..

[38] Huang da W, Sherman BT, Lempicki RA (2009). Systematic and integrative analysis of large gene lists using DAVID bioinformatics resources. Nat. Protoc..

[39] Huang da W, Sherman BT, Lempicki RA (2009). Bioinformatics enrichment tools: paths toward the comprehensive functional analysis of large gene lists. Nucleic Acids Res..

[40] van Iersel MP (2008). Presenting and exploring biological pathways with PathVisio. BMC Bioinforma..

[41] Kutmon M (2015). PathVisio 3: an extendable pathway analysis toolbox. PLoS Comput. Biol..

[42] Bogan RL, Murphy MJ, Stouffer RL, Hennebold JD (2008). Systematic determination of differential gene expression in the primate corpus luteum during the luteal phase of the menstrual cycle. Mol. Endocrinol..

[43] Xu J (2016). Anti-Mullerian hormone is produced heterogeneously in primate preantral follicles and is a potential biomarker for follicle growth and oocyte maturation in vitro. J. Assist. Reprod. Genet..

[44] Chaffin CL, Dissen GA, Stouffer RL (2000). Hormonal regulation of steroidogenic enzyme expression in granulosa cells during the peri-ovulatory interval in monkeys. Mol. Hum. Reprod..

[45] Zhou X (2015). Prosaposin facilitates sortilin-independent lysosomal trafficking of progranulin. J. Cell. Biol..

[46] Cheng, M. H. & Jansen, R. P. A jack of all trades: the RNA-binding protein vigilin. *Wiley Interdiscip. Rev. RNA* **8**, 1448 (2017). 10.1002/wrna.1448 28975734

[47] Takahashi N (2012). Necrostatin-1 analogues: critical issues on the specificity, activity and in vivo use in experimental disease models. Cell death & Dis..

[48] Zhou W, Yuan J (2014). Necroptosis in health and diseases. Semin. Cell. Dev. Biol..

[49] Grootjans, S., Vanden Berghe, T. & Vandenabeele, P. Initiation and execution mechanisms of necroptosis: an overview. *Cell Death Differ.* **24**, 1184 (2017). 10.1038/cdd.2017.65 PMC5520172 28498367

[50] Abraham MC, Lu Y, Shaham S (2007). A morphologically conserved nonapoptotic program promotes linker cell death in *Caenorhabditis**elegans*. Dev. Cell..

[51] Wang SW (2015). Regulation of ceramide generation during macrophage apoptosis by ASMase and de novo synthesis. Biochim. Et. Biophys. Acta (BBA) - Mol. Cell Biol. Lipids.

[52] Parisi LR, Li N, Atilla-Gokcumen GE (2017). Very long chain fatty acids are functionally involved in necroptosis. Cell Chem. Biol..

[53] Domijan AM, Zeljezic D, Milic M, Peraica M (2007). Fumonisin B(1): oxidative status and DNA damage in rats. Toxicology.

[54] Vandenabeele P, Riquet F, Cappe B (2017). Necroptosis: (last) message in a bubble. Immunity.

[55] Santana P (1995). Ceramide mediates tumor necrosis factor effects on P450-aromatase activity in cultured granulosa cells. Endocrinology.

[56] Witty JP, Bridgham JT, Johnson AL (1996). Induction of apoptotic cell death in hen granulosa cells by ceramide. Endocrinology.

[57] Liao D (2014). Necrosulfonamide inhibits necroptosis by selectively targeting the mixed lineage kinase domain-like protein. MedChemComm.

[58] Sawai H, Ogiso H, Okazaki T (2015). Differential changes in sphingolipids between TNF-induced necroptosis and apoptosis in U937 cells and necroptosis-resistant sublines. Leuk. Res..

[59] Sentelle RD (2012). Ceramide targets autophagosomes to mitochondria and induces lethal mitophagy. Nat. Chem. Biol..

[60] Devoto L, Kohen P, Munoz A, Strauss JF (2009). Human corpus luteum physiology and the luteal-phase dysfunction associated with ovarian stimulation. Reprod. Biomed. Online.

[61] Fabregat A (2018). The reactome pathway knowledgebase. Nucleic Acids Res..

