# Supplementary material for: Necroptosis in primate luteolysis: a role for ceramide
Source: Cell Death Discov. 2019 Feb 11;5:67. doi: 10.1038/s41420-019-0149-7 (PMC6370808; doi:10.1038/s41420-019-0149-7)
Supplement: Supplementary file 1 — supplementary files [file 41420_2019_149_MOESM1_ESM.docx]

**Supplement File**

***Supplement Fig. 1: Pathway illustration of known reactions of sphingolipid metabolism and the ceramide salvage pathway.***

Metabolites are shown in rectangular boxes with rounded corners and proteins in rectangular boxes colored according to the log_2_ fold change between day 5 and day 2. Proteins were colored with the shown gradient according to their log_2_ fold change. Proteins with no significant change in abundancies are shown in grey.

***Supplement Fig. 2: Illustration of ceramide salvage pathway associated genes/proteins generated by transcriptomic/proteomic analysis.***

LFQ values and log_2_ transformed transcriptome data for specified proteins/genes are shown in column diagrams. In most cases, the tendency was the same for proteomic and transcriptomic data. P-values and fold changes are shown in Supplement Tab. 1 and p < 0.05 is marked with an asterisk (*). Proteomic data, n = 5; transcriptomic data, n = 8. Error bars indicate SEM.

***Supplement Fig. 3: Confocal microscopy images of immunostained GCs and Western blot results showing actions of NSA***

(a) GCs, cultured on glass coverslips were fixed on day 2 or 5 of culture, or stimulated with NSA (20 µM, 72 h) before fixation. Immunocytochemical staining using anti-ceramide and anti-golgin97 antibodies revealed different intensities and loci due to culture time and stimulation. Controls using mouse IgG and IgM are shown in insets of the first column. Three independent experiments were conducted, and representative pictures are shown. Scale bars indicate 50 µm.

(b, c) Western Blot of GCs stimulated for 72 h either with (b) the solvent control, (b, c) 50 µM C2-CER alone or (c) in combination with 20 µM NSA showed phosphorylation of MLKL at T357/S358, and oligomerization. In all groups, monomeric pMLKL(T357/S358) bands at <55 kDa and octameric pMLKL(T357/S358) at >250 kDa were evident. The Western Blots were evaluated by quantification of 5 independent experiments per group. Intensity of the pMLKL(T357/S358) bands (monomeric + oligomeric) were normalized to the MLKL band. Representative blots are shown. (b, c) paired Student’s *t*-test was conducted to evaluate statistical significance (*p < 0.05); means and SEM are shown.

***Supplement Fig. 4: Confocal microscopy images of immunostained GCs showing actions of FB1 and Nec1s.***

(a) GCs, cultured on glass coverslips were fixed on day 2 (left column) or day 5 (middle column) of culture, with or without stimulation by FB1 (0.5 µM, 72 h, right column) before fixation. Controls using rabbit IgG and mouse IgM are shown in insets of the first row. (b) In another experiment GCs were cultured the same way but stimulated with Nec-1s (20 µM, 72 h, right column) instead of FB1. Immunocytochemical staining using anti-ceramide and anti-golgin97 antibodies revealed different intensities and cellular localization, depending on culture time and stimulation. Two independent experiments were performed and representative pictures are shown. Scale bars indicate 25 µm (a) and 50 µm (b).

***Supplement Tab. 1: The levels of proteins and genes (mRNA) involved in ceramide salvage pathway, which were upregulated after 5 days of culture in human IVF-derived GCs and in the late stage macaque CL, respectively.***

The p-values resulted from Student’s *t-*test with Welch’s correction analysis of log_2_ fold change values of protein and mRNA expression data of cultured GCs and timed macaque CL.

***
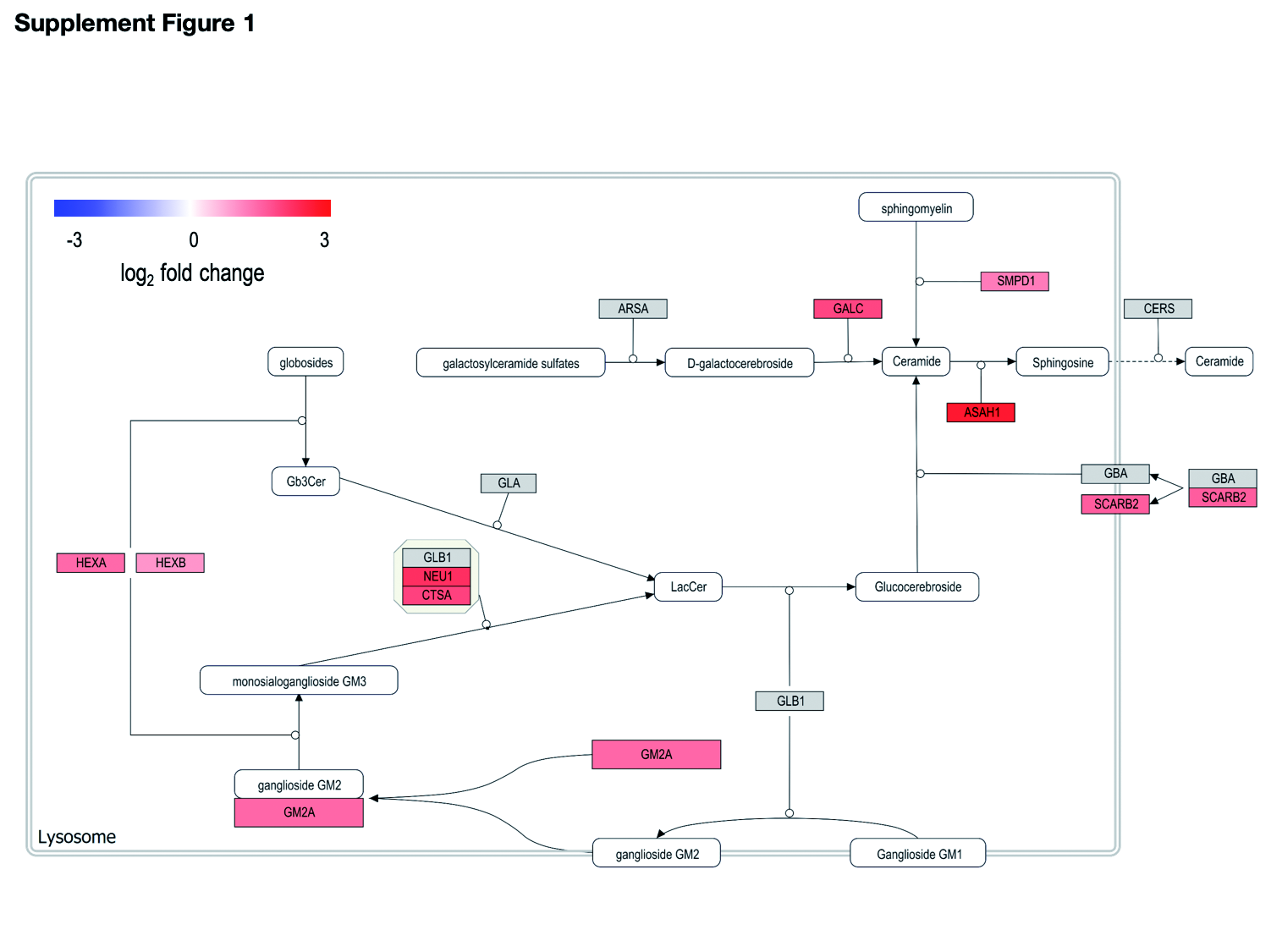
*Supplement Figure 1**


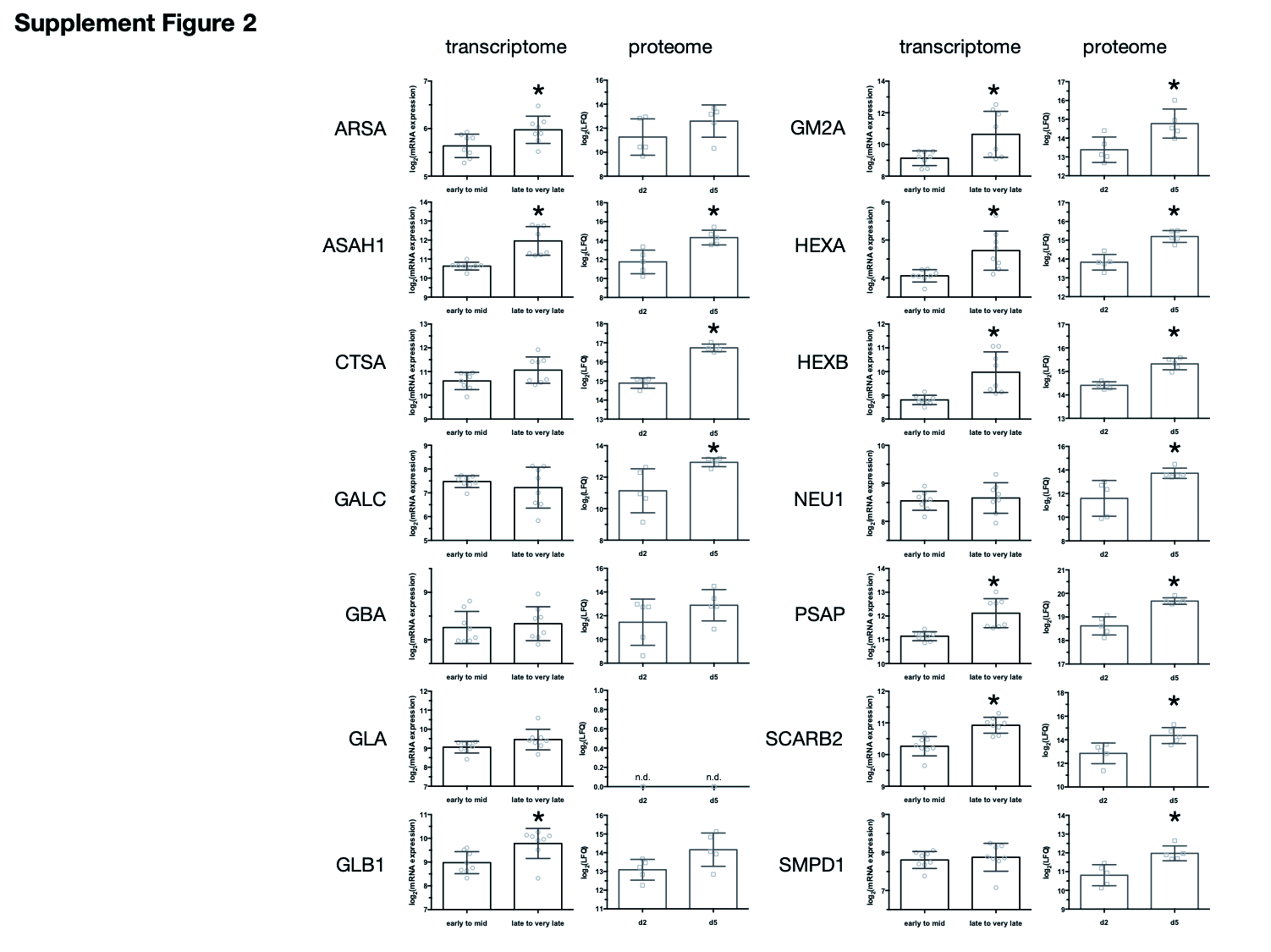
**Supplement Figure 2**


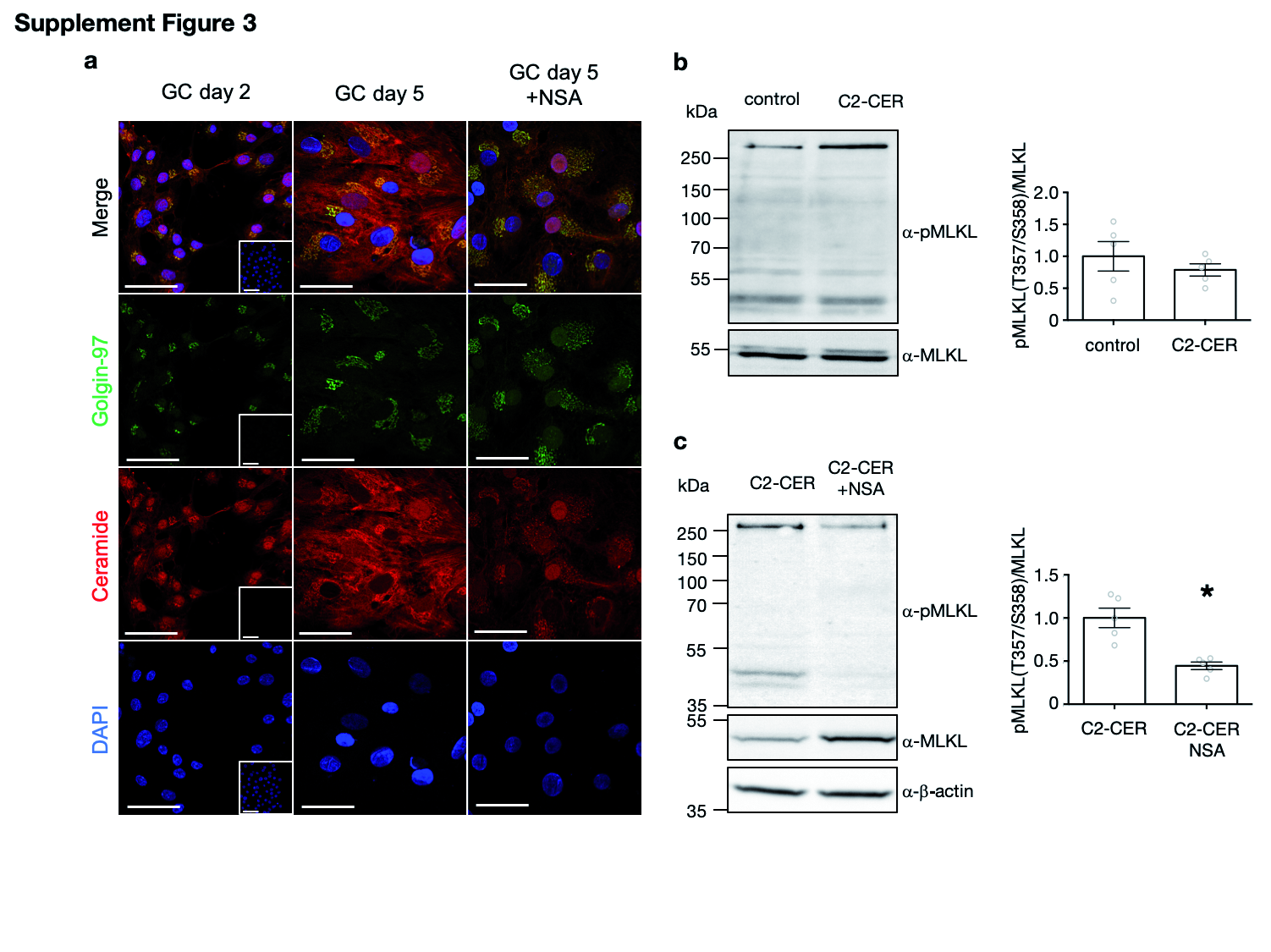
**Supplement Figure 3**

**
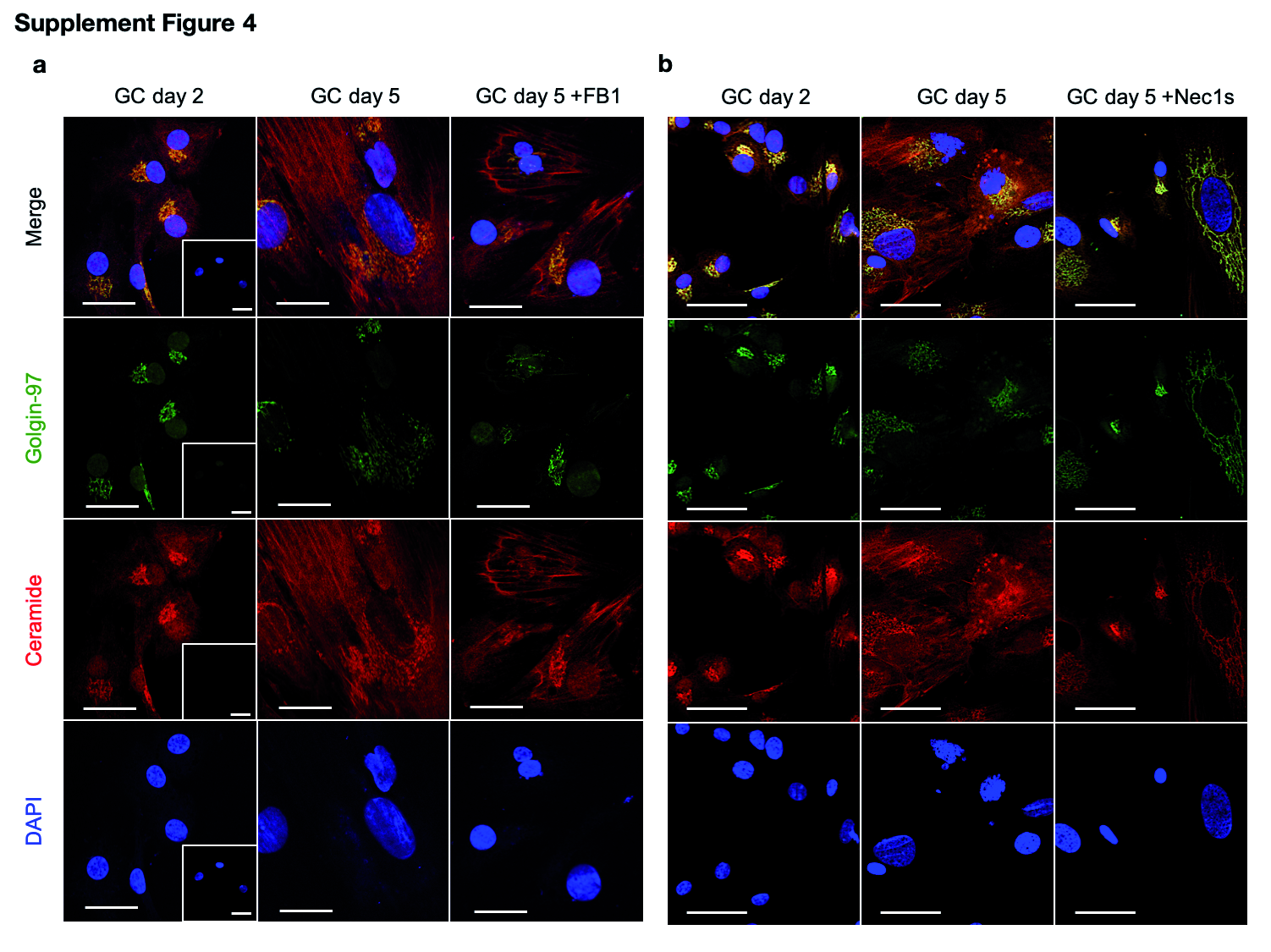
Supplement Figure 4**

***Supplement Table 1***

|  |  | protein levels (GC d5 vs. d2) | | mRNA levels (late vs. early CL) | |
| --- | --- | --- | --- | --- | --- |
| gene name | protein name | p value | log2 FC | p value | log2FC |
| ARSA | arylsulfatase A | 0.181 | 1.329 | 0.024 | 0.338 |
| ASAH1 | acid ceramidase | 0.006 | 2.568 | 0.001 | 1.326 |
| CTSA | cathepsin A | 3.72E-06 | 1.848 | 0.071 | 0.456 |
| GALC | galactocerebrosidase | 0.043 | 1.809 | 0.439 | -0.252 |
| GBA | glucosylceramidase | 0.217 | 1.427 | 0.649 | 0.081 |
| GLA | a-galactosidase A | n.d. | n.d. | 0.091 | 0.399 |
| GLB1 | b-galactosidase | 0.058 | 1.073 | 0.011 | 0.807 |
| GM2A | GM2 ganglioside activator | 0.017 | 1.389 | 0.014 | 1.510 |
| HEXA | hexosaminidase subunit a | 4.81E-04 | 1.369 | 0.004 | 0.662 |
| HEXB | hexosaminidase subunit b | 2.81E-04 | 0.911 | 0.002 | 1.168 |
| NEU1 | sialidase 1 | 0.032 | 2.124 | 0.661 | 0.075 |
| PSAP | prosaposin | 0.002 | 1.056 | 0.001 | 0.967 |
| SCARB2 | lysosomal membrane protein 2 | 0.017 | 1.510 | 0.001 | 0.661 |
| SMPD1 | acid sphingomyelinase | 0.006 | 1.166 | 0.646 | 0.071 |
